# Optimal Design and Development of Magnetic Field Detection Sensor for AC Power Cable

**DOI:** 10.3390/s24082528

**Published:** 2024-04-15

**Authors:** Yong Liu, Yuepeng Xin, Youcong Huang, Boxue Du, Xingwang Huang, Jingang Su

**Affiliations:** 1 The Key Laboratory of Smart Energy and Information Technology of Tianjin Municipality, Tianjin University, Tianjin 300072, China; 2022234338@tju.edu.cn (Y.X.); duboxue@tju.edu.cn (B.D.); 2 The Key Laboratory of Smart Grid of Ministry of Education, Tianjin University, Tianjin 300072, China; 3 The State Grid Fujian Electric Power Research Institute, Fuzhou 350007, China; hyoucong@163.com; 4 The State Grid Hebei Electric Power Research Institute, Shijiazhuang 050011, China; huangxingwang@tju.edu.cn (X.H.); sujingang@tju.edu.cn (J.S.)

**Keywords:** single-core AC high-voltage cable, magnetic field sensor, optimal design, magnetic core, coil, amplifying circuit

## Abstract

The state detection of power cables is very important to ensure the reliability of the power supply. Traditional sensors are mostly based on electric field detection. The operation is complex, and its efficiency needs to be improved. This paper optimizes the design and development of the magnetic field detection sensor for AC power cables. First, through the establishment of the magnetic field sensor model, it is determined that permalloy is the material of the magnetic core, the optimal aspect ratio of the magnetic core is 20, and the ratio of coil length to core length is 0.3. Second, the coil-simulation model is established, and it is determined that the optimal number of turns of the coil is 11,000 turns, the diameter of the enameled copper wire is 0.08 mm, and the equivalent magnetic field noise of the sensor is 0.06 pT. Finally, the amplifying circuit based on negative magnetic flux feedback is designed, the sensor is assembled, and the experimental circuit is built for the sensitivity test. The results show that the sensitivity of the magnetic field sensor is 327.6 mV/μT. The sensor designed in this paper has the advantages of small size, high sensitivity, ease of carry, and high reliability.

## 1. Introduction

Due to the characteristics of stable transmission performance, good insulation performance, high temperature resistance, easy laying, and simple operation and maintenance, power cables have been widely used in the power grid. However, the layout of power cables is mostly in the form of tunnels, channels, pipes, and direct burial [1]. The layout of these cables brings much inconvenience to the prediction and maintenance of power-cable faults [2]. The insulation medium of the cable is easily made damp in the ground, and the ability of ventilation and heat dissipation is poor. This will reduce the insulation performance of the cable. In addition to natural reasons, some artificial reasons, such as artificial destruction of the cable and installation of non-standard operation, will also cause cable failure [3]. Therefore, it is very important to carry out the detection of cable aging, defects, and faults.

The cable detection methods are mainly divided into offline detection methods and online detection methods. Offline detection methods include dielectric loss factor test, partial discharge test, DC voltage test, AC voltage test, etc. Online monitoring methods include the DC superposition method, DC component method, AC superposition method, etc. [4,5,6]. All these methods are based on the detection of current and discharge phenomena caused by the electric field, which have two obvious shortcomings. On the one hand, in the cable test, it is necessary to add measurement equipment and modify the grounding system, which will destroy the operating state of the cable system. On the other hand, the detection signal is easily interfered with by external environmental factors, and the detection signal is easily overwhelmed. Therefore, new sensors and detention methods are increasingly required for the monitoring of the cable system.

Because of its own nature and working conditions, the power cable will produce a magnetic field around its daily work. As magnetic field sensors have the advantages of high accuracy, high sensitivity, and wide detection depth [7], it is proposed to apply the magnetic field detection method in the monitoring and evaluation of power-cable systems. By combining magnetic field sensors with RFID technology, Shao et al. provide a promising approach for wireless cable current detection and a potential design for a universal wireless passive sensing system. The current measurement ranges at 50 Hz and 60 Hz are both 5–17.5 A, the maximum measurement errors are 1.83% and 1.77% respectively, and the maximum wireless distance is 5.2 m. It can be intuitively seen that this device has a greater advantage in detection distance, but its large size makes it inconvenient to carry for daily operation and maintenance [8]. Suo et al. proposed a three-phase four-wire phase current non-contact measurement method based on magnetic field decoupling calculation to solve the problem that traditional current measurement methods cannot measure the phase current of four-core cables. Through experimental verification, it is determined that the overall measurement amplitude error of this design is less than 2.2%, and the phase-angle error is less than 3°. This technology can better solve the problem that traditional inductance measurement devices could not measure phase current in the past, thereby achieving random and real-time measurement. However, the experiments of this design were all conducted under the conditions of a current less than 5 A and a frequency of 50 Hz. Whether the measurement accuracy of a large current can maintain a high standard still needs to be verified [9]. Zhu et al. proposed an event-driven non-intrusive multi-core cable current monitoring based on magnetic field sensors. The team designed a non-invasive sensing device using a circular sensor array to measure current in multi-core cables. The team verified the feasibility of the method through laboratory experiments and field-test results. In multi-core cable current monitoring, the average current relative error can ultimately be less than 1%. This design achieves excellent monitoring of AC cables by being installed on the cable body structure [10]. Yang et al. proposed a new type of arc-shaped magnetic core. The curved iron core designed by the team fits the surface of the cable, which can effectively increase the magnetic field strength in the area where the FSMFEH is located. In addition, based on theoretical analysis, the team proposed the optimal coil turns design method for the arc-shaped core FSMFEH and conducted experimental verification [11].

It can be seen that most of the existing magnetic field sensors for power-cable detection remain at the theoretical stage up to now. Only a few sensors can achieve high-precision detection of cables or locations with one or more specific voltage levels. This also makes it difficult for the detection accuracy and stability of existing research to meet engineering standards in the field of power engineering. At the same time, most of these currently existing sensors are fixed on the cable structure for a long time and perform long-term detection. However, in actual power engineering, the information obtained by maintenance personnel from the dispatch center can only determine the line with the problem. For engineering sites, maintenance personnel are in urgent need of a device that can quickly find the location of specific cable defects. Therefore, this paper optimizes the design and development of the magnetic field detection sensor for AC power cables. The influencing factors of coil quality and noise were analyzed to determine the parameters of the coil. In this paper, the amplifying circuit based on negative flux feedback is designed by using Multisim, and the hardware of the sensor is designed and assembled. After that, this paper sets up an experimental circuit for sensitivity testing, and the experimental results show that the magnetic field sensor designed in this paper can achieve high sensitivity. The sensor designed in this paper has the advantages of small size, high sensitivity, ease of carry, and high reliability, which is of great significance for maintaining the stable operation of power cables and finding and repairing power-cable faults in time, which is of great significance for maintaining the stable operation of power cable, promptly discovering and repairing power cable faults.

## 2. Principle of Magnetic Field Sensor

Magnetic field measurement involves a wide range, and there are many measurement methods corresponding to different physical principles. Currently, there is a wide range of measurement methods in industrial applications, which can be roughly divided into the magnetic torque method, fluxgate method, electromagnetic induction method, electromagnetic effect method, and magneto-optical effect method, as shown in Table 1 [12,13,14,15,16].

The magnetic field around the power cable is a medium-low intensity magnetic field [17]. For the method of measuring medium-low intensity magnetic fields, the magnetic saturation method is suitable for low-frequency or constant magnetic fields, while the magneto-optical effect method requires very-high instrument manufacturing processes. As a kind of electromagnetic induction mode, an inductive sensor has the advantages of a simple working principle, simple manufacture and installation, relatively small volume, and much lower production cost than the magneto-optic effect. It can be seen that the magnetic field measured by the electromagnetic induction is less than 0.1 T, which can be used to measure alternating magnetic fields and weakly varying magnetic fields. Therefore, this paper chose the electromagnetic induction method as the basic principle of the sensor.

**Figure 1:**
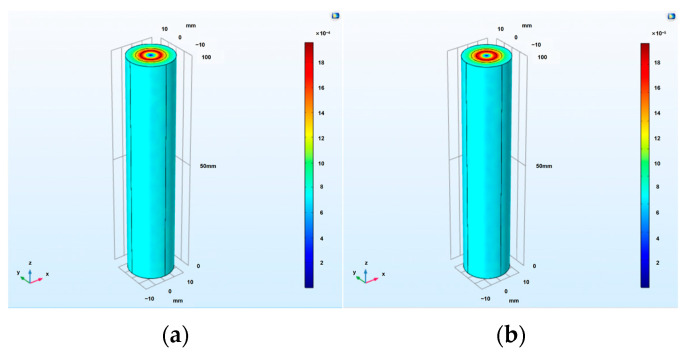
Cable magnetic field distribution. (**a**) Magnetic field distribution of single−core cable when the effective value is 200 A AC current. (**b**) Magnetic field distribution of single−core cable when the effective value is 50 A AC current.

A single-phase cable model was established by using COMSOL. AC currents with effective values of 200 A and 50 A were applied to the cable, respectively, and the magnetic field intensity distribution diagram of the cable is shown in Figure 1. It can be seen that, when an alternating current with an effective value of 200 A was applied to the cable, the magnitude of the outermost magnetic field strength of the cable was 10^−4^. When an alternating current with an effective value of 50 A was applied to the cable, the magnitude of the outermost magnetic field strength of the cable was 10^−5^. According to the COMSOL simulation results, it can be seen that there is a weak magnetic field around the power cable when it is energized, and its size is within the measurement range of the magnetic field sensor (sensor based on the principle of electromagnetic induction). Therefore, the electromagnetic induction method is chosen as the basic designing principle of the magnetic field sensor [18].

**Figure 2:**
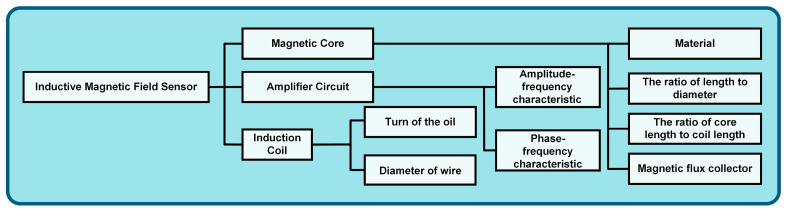
Structure diagram of designing the magnetic field sensor.

The design of the magnetic field sensor in this paper is divided into three parts: magnetic core, induction coil, and amplifier circuit, as shown in Figure 2.

## 3. Design of Magnetic Core

The design of the magnetic core mainly considers two aspects: the selection of the magnetic core material and the determination of the geometric size of the magnetic core.

### 3.1. Selection of Magnetic Core Material

The ferromagnetic materials can reach saturation values under an external small magnetic field. The magnetic susceptibility is not only positive but can also reach the order of 10–10^6^, so its magnetic permeability is also very high. The inductive magnetic sensor uses a magnetic core structure in order to improve the initial sensitivity by using the magnetic core’s higher magnetic permeability than air; so, a ferromagnetic material with high magnetic permeability is a good choice. 

**Figure 3:**
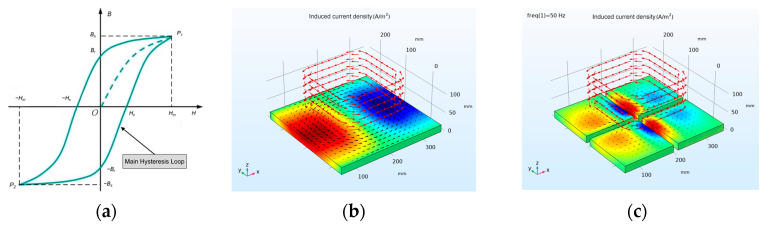
Simulation diagram of eddy−current generation: (**a**) Hysteresis loop of ferromagnetic materials. (**b**) Induced current density in conductor during 50 Hz. (**c**) Induced current density in conductor after block during 50 Hz.

During the magnetization process of the ferromagnetic material, the relationship between the magnetic induction intensity *B* in the material and the external magnetic field intensity *H* is shown in Figure 3a. The hysteresis loop reflects the properties of ferromagnetic materials. When the hysteresis-loop area is large, the magnetic field energy consumed by magnetization in the alternating magnetic field is large, and the material will also heat up during repeated magnetization. A ferromagnetic material with larger hysteresis-loop areas is called a hard magnetic material, and materials with smaller hysteresis-loop areas are called soft magnetic materials. For the sensor core, the increase in core temperature during operation will reduce the magnetic permeability of the core, thereby reducing the sensor’s induced voltage. Therefore, when designing the sensor, the heat generated by the magnetic core material during operation should be minimized, which requires the selected material to have a smaller area of the hysteresis loop. Therefore, soft magnetic materials should be used when selecting magnetic core materials.

Loss is an important index to evaluate the properties of a magnetic material. There are two main ways of core loss: hysteresis loss and eddy-current loss. The hysteresis loss is caused by the energy consumed by changing the direction of the magnetic moment in the domain. Eddy-current loss is usually divided into two parts: one is called classical eddy-current loss and the other is called residual loss. 

A model for simulating eddy-current generation is established in COMSOL, as shown in Figure 3b, where above the aluminum plate is a multi-turn coil. The number of turns of the coil is 2472 turns, and a current with an amplitude of 1 A and a frequency of 50 Hz is passed into each turn. The current is sinusoidal. When an AC is passed through the coil, an induced eddy current and an induced magnetic field will be generated on the aluminum plate. The aluminum plate in Figure 3b is divided into four parts and then simulated, and the eddy-current effect in the conductor is shown in Figure 3c.

The size of the magnetic core determines the sensitivity of the sensor. The material of the magnetic core plays a role in the converging magnetic flux and increasing the induced voltage. The application frequency band of the induction magnetic sensor to be designed is 10–500 Hz, the operating temperature is −40–125 °C, and the magnetic induction intensity is less than 1 mT. Therefore, the requirements for the magnetic core are as follows: the saturation magnetic induction strength can be on the order of T; the low coercive force is to reduce hysteresis loss; the permeability has high stability to temperature and frequency; the low resistivity is to reduce eddy-current losses; the frequency range is moderate, including the frequency band of the sensor; and the relative permeability is above 10^4^.

Table 2 gives the typical parameters for several materials [19,20,21,22,23]. The resistivity of Permalloy is not much different from that of amorphous or nanocrystalline alloys. However, Permalloy has lower coercive force and high saturation magnetic field strength. Compared to amorphous alloys and nanocrystalline alloys, Permalloy thin ribbons are softer and easier to make laminated magnetic cores, and they are cheaper. Therefore, Permalloy was selected as the core material.

### 3.2. Determination of Magnetic Core Parameters

**Figure 4:**
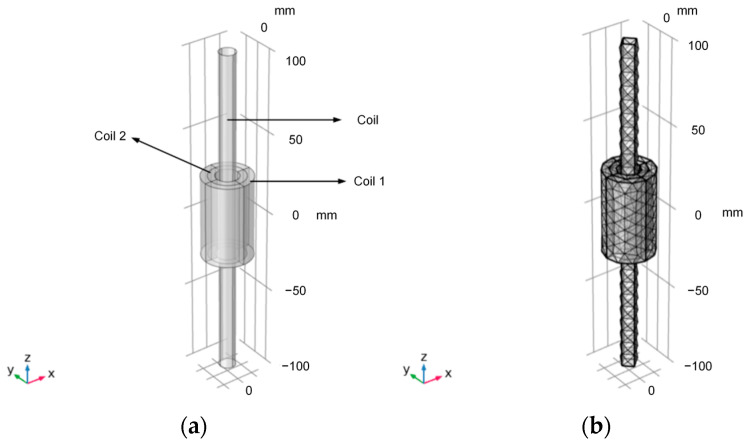
The geometric model and the meshing diagram of the magnetic core and coil: (**a**) the geometric model of the magnetic core and coil; (**b**) the meshing diagram of the model.

As shown in Figure 4, the model of the magnetic core and coil is established by COMSOL. This model considers the nonlinear *B-H* curve of the magnetic core and calculates the spatial distribution of magnetic and electric fields, the magnetic saturation effects, and the transient response. Coil 1 and coil 2 are simulated using the “coil” feature. The external circuit of coil 1 and coil 2 is connected to the AC voltage source and the resistor through the “circuit” interface. The “coil geometry analysis” research step is used to calculate the current in the coil. By adding a “transient” study, the voltage and current in coil 1 and coil 2 are determined. Coil 1 is connected to an external AC power source and a resistor, and its role is to generate a changing magnetic field. Due to the generation of the AC magnetic field, an induced potential will be generated across coil 2. Coil 2 is externally connected to a resistor, and the output voltage of the coil is represented by the voltage across the resistor. The magnetic core can limit the magnetic field to the middle of the coil to the greatest extent [24].

**Figure 5:**
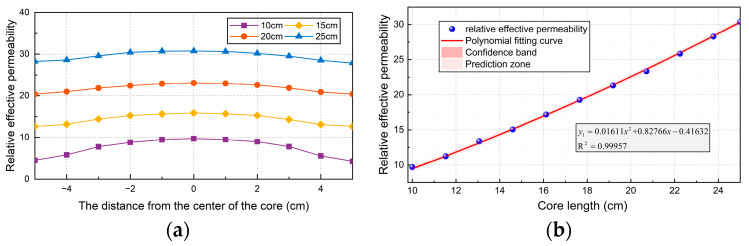
The influence of magnetic core length on the performance of the sensor: (**a**) The relationship between the relative effective permeability of each length of core and its position in the core; (**b**) the relationship between relative effective permeability and magnetic core length.

The influence of core length on the performance of the sensor is studied by establishing the simulation model of four cylindrical cores. The radius of the core is 2 cm, and the length is 10, 15, 20, and 25 cm, respectively. The relationship between the relative effective permeability of each length of the core and its position in the core is shown in Figure 5a. Figure 5b shows the relationship between the relative effective permeability of the geometric center of the core and the length of the core.

When the magnetic material is magnetized by the external magnetic field, in addition to the magnetic permeability of the material itself, there is also a demagnetizing field in the material itself, so *B ≠ μH_out_*. In engineering practice, the demagnetization field in the magnetic core is not uniform and constant. This paper assumes that the demagnetizing field is uniform, and its expression is [25]:

(1) $$H_{d}=\frac{M\cdot N_{d}}{\mu_{0}}$$

where *μ*_0_ is the vacuum permeability, and *N_d_* is the demagnetizing factor. The equivalent formula of the magnetic induction intensity *B* in the material is:

(2) $$B=\mu H=\mu_{0}H+M$$

From this, we can get:

(3) $$H_{out}=\left[N_{d}\left(\mu -\mu_{0}\right)/\mu_{0}+1\right]H$$

The factor that affects the size of the demagnetizing field is called the demagnetizing factor *N_d_*. The demagnetizing factor is a function of the material position, and its expression is shown in Formula (4). Where *μ* is the surface permeability of the magnetic core, *m* is the aspect ratio of the magnetic core, and *c* is the shape coefficient of the magnetic core (*c* is 0.8). Therefore, the demagnetizing field *H_d_* within the material is also related to the position of the material and is also a function of the position of the material.

(4) $$N_{d}=\frac{4\pi }{m^{2}\left(1-\frac{c}{3}\right)}\left(ln1.2m-1\right)$$

In fact, in the field of practical engineering, it is difficult to determine the specific function of the demagnetizing field or demagnetizing factor and its position in the material. Therefore, the concept of effective magnetic permeability *μ_app_* is proposed to simplify the study of the internal magnetic field of materials. If the effective magnetic permeability is defined as the ratio of the magnetic induction intensity *B* within the material to the external magnetic field intensity *H_out_*, then the relative effective magnetic permeability *μ_app_* is:

(5) $$\mu_{app}=\frac{B/H_{out}}{\mu_{0}}=\frac{\mu }{\left(\mu -\mu_{0}\right)N_{d}+\mu_{0}}$$

The material of the core needs to have high permeability, so *μ*/*μ*_0_ is much greater than 1. The equation can be expressed as:

(6) $$\mu_{app}=\frac{1}{N_{d}}$$

**Figure 6:**
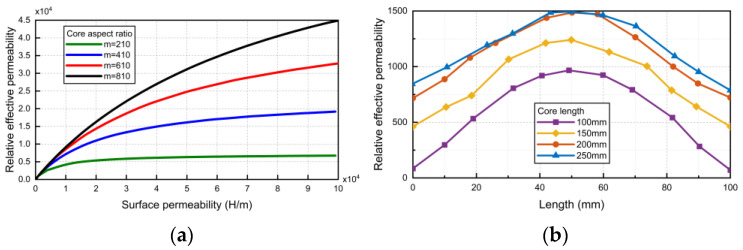
Effect of length–diameter ratio of magnetic core on sensor performance: (**a**) the relationship between effective permeability, aspect ratio, and surface permeability of magnetic core; (**b**) effect of length–diameter ratio of magnetic core on effective permeability.

Figure 6a shows the relationship between the effective permeability, aspect ratio, and surface permeability of the core. Through comparison, it can be determined that permalloy is the best choice as the core material of the sensor.

The sensor simulation model is established in COMSOL. When the core diameter is set to 10 mm, the length of the core is constantly changing, as shown in Figure 6b. It can be seen that the longer the core is, the greater the relative effective permeability is. When it increases to 200 mm, the effective permeability no longer increases with the increase of core length. Therefore, the optimum aspect ratio of the core is 20.

**Figure 7:**
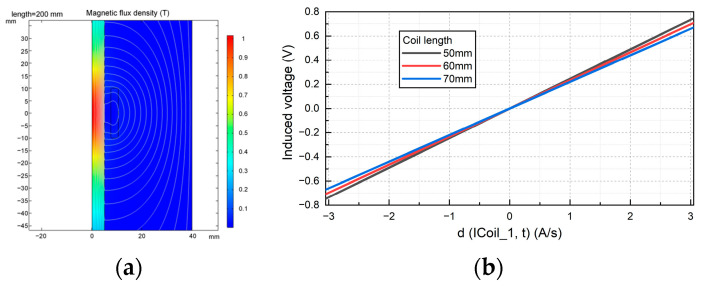
Effect of the ratio of coil length to core length on the performance of the sensor: (**a**) magnetic field distribution of magnetic core; (**b**) effect of coil length on induced voltage of magnetic field sensor.

When the core length is equal to 200 mm, the magnetic field distribution in the core is shown in Figure 7a. The results show that the magnetic induction intensity at the center of the core is the highest and the sensitivity is the highest. Therefore, to avoid the edge effect, the length of the coil should be less than the length of the magnetic core. However, the length of the coil should not be too short, because the length of the copper wire with the same number of coils will be longer, thus making the DC impedance higher. The longer the length of the coil, the greater the noise and the greater the interference to the output signal. Therefore, it is necessary to choose an appropriate coil length to ensure the excellent performance of the sensor.

The effect of the ratio of coil length to core length on the inductive magnetic field sensor is shown in Figure 7b. The simulation results show that, to obtain a larger induction voltage, the induction coil should be distributed near the center of the magnetic core. Taking a magnetic core with a diameter of 10 mm and a length of 200 mm as an example, at a distance from the center of the core ±33 mm, the magnetic field intensity of the core is reduced by about 7% to the maximum value, and the sensitivity of the sensor also meets the requirements. Therefore, the induction coil is distributed at the center of the magnetic core, and the ratio of the length of the magnetic core to the length of the induction coil is 0.3.

When making the magnetic core for the sensor, in addition to its electromagnetic properties, it is also necessary to consider its mechanical properties. In practice, considering the manufacturing process of the magnetic core and the mechanical strength of the material, the ratio that is too small between the cross-sectional area and the length will make the magnetic core prone to breakage during production and use. The sensor studied in this paper is generally used to detect power cables in the field. An excessively long magnetic core length will be inconvenient for inspection personnel to carry. For the above reasons, the aspect ratio of the magnetic core will be limited. In order to further improve the effective permeability of the core, it is necessary to change the structure of the core. Adding a flux collector to the core is a method to change the structure of the core so as to improve the effective permeability of the core. 

**Figure 8:**
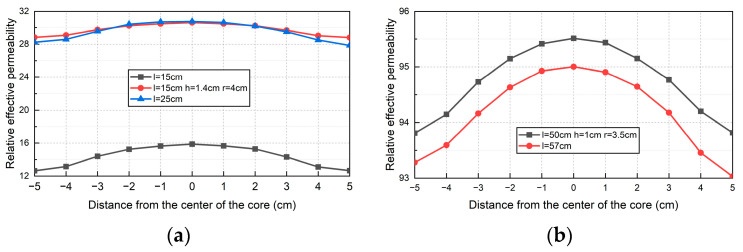
Effect of flux collector on the performance of sensor: (**a**) effect of adding flux collector and increasing core length on effective permeability of magnetic core; (**b**) relative effective permeability of magnetic core with or without flux collector.

In this paper, for different magnetic flux collectors, a cylinder with the same radius is selected as the magnetic core (*r* = 2 cm), and magnetic flux collectors are added at both ends of the magnetic core. In order to explore the effect of adding magnetic flux collectors, two disc-shaped magnetic flux collectors (radius: *r* = 4 cm, thickness: *h* = 1.4 cm) were added to both ends of the magnetic core. The material used was the same lossless soft iron material as the magnetic core. This paper compared the relative effective magnetic permeability simulation results obtained after adding flux collectors with the simulation results without adding magnetic flux collectors. The radius of the two magnetic cores without adding flux collectors is 2 cm, and the length is 15 cm and 25 cm, respectively. The curves are shown in Figure 8a. It can be seen from Figure 8a that, after adding the flux collector, the relative effective magnetic permeability of a 15 cm long magnetic core in a linear region 5 cm away from the geometric center along the axial direction increases by an average of 15.38. These data are 107% higher than the situation without adding a magnetic flux collector, achieving a similar effect when the core length is increased to 25 cm. If the core length is increased to 25 cm, 66.67% additional material needs to be added. At this time, if a magnetic flux collector is added, an additional 74.66% of material needs to be added. Obviously, the effect of adding a flux collector at this time is not as good as increasing the core length.

This article increased the length of the magnetic core to 50 cm and added magnetic flux collectors (radius: *r* = 3.5 cm, thickness: *h* = 1 cm) at both ends of the magnetic core. Comparing the simulation results of this model with the magnetic field simulation results of a cylindrical core (*l* = 57 cm, *r* = 2 cm), the relative effective magnetic permeability curve was obtained, as shown in Figure 8b. After adding the magnetic flux collector, the relative effective permeability of the central area of the axis of the core with a length of 50 cm is higher than that of the core with a length of 57 cm. Adding a magnetic flux collector increased the material and weight by 12.25% compared to the original without adding it. However, when increasing the length to 57 cm, the core required 14% more material and weight. At this time, adding a magnetic flux collector has a better effect on increasing the relative effective magnetic permeability of the magnetic core than increasing the length of the magnetic core.

A preliminary conclusion can be drawn from Figure 8 that, when the length of the core is small or the length–diameter ratio of the core is small, the efficiency of adding the flux collector to increase the effective permeability of the core is not high. When the length of the core reaches a certain value, the efficiency of adding a flux collector is higher.

**Figure 9:**
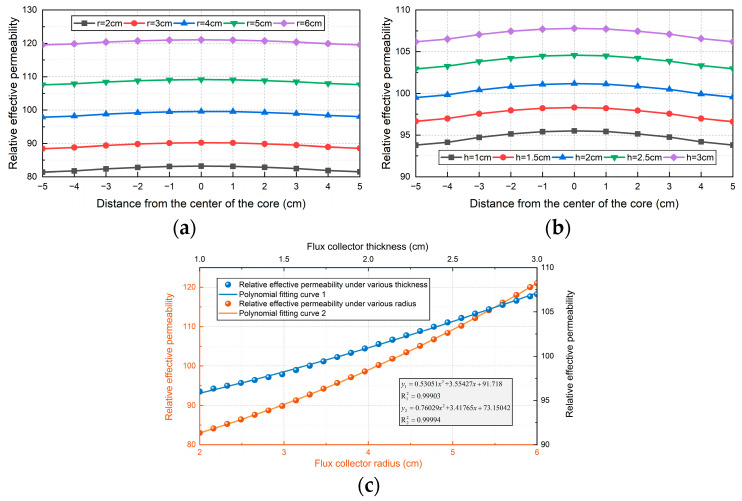
Effect of flux collector on the performance of sensor: (**a**) relative effective permeability of magnetic core of flux collector with different thickness; (**b**) relative effective permeability of magnetic core of flux collector of each radius; (**c**) polynomial fitting curve.

- When the radius *r* of the flux collector is constant and the thickness *h* is different, the relative effective permeability of the core axis is shown in Figure 9a. With the increase of the thickness of the flux collector, the relative effective permeability of the core will increase. As shown in Figure 9d, the relative effective permeability of the core increases with the increase of the thickness of the trap;
- When the thickness *h* of the flux collector is a certain value and the radius *r* is different, the relative effective permeability distribution is shown in Figure 9b. We can see that, when *r* increases at equal intervals, the rate of increase of the relative effective permeability will gradually increase. The fitting curve is shown in Figure 9c; the relative effective permeability increases in a parabola with the increase of the radius of the collector.

The inductive magnetic field sensor designed in this paper is mainly used in portable cable-detection devices. It is necessary to ensure the portability of the equipment by not being too large in size. Therefore, according to the above discussion and research, since the effect of adding the magnetic flux collector is not significant when the magnetic core length is small, the magnetic field sensor designed in this paper does not have magnetic flux collectors at both ends of the magnetic core.

## 4. Design of Induction Coil

### 4.1. Resistance, Inductance, and Capacitance of Induction Coil

**Figure 10:**
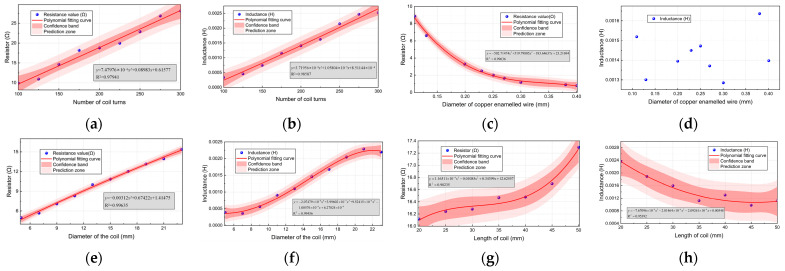
The experiment verifies the diagram: (**a**) the effect of the number of turns of the coil on the resistance; (**b**) the effect of the number of turns of the coil on the inductance; (**c**) the effect of copper enameled wire diameter on resistance; (**d**) the effect of copper enameled wire diameter on inductance; (**e**) the effect of coil diameter on the resistance; (**f**) the effect of coil diameter on inductance; (**g**) the effect of coil length on the resistance; and (**h**) the effect of coil length on inductance.

As shown in Figure 10, the effects of coil turn, copper enameled wire diameter, coil diameter, and coil length on coil resistance and inductance are analyzed by experiment.

- The number of turns of the coil and the diameter of the copper enameled wire will affect the coil resistance, and the length of the coil will not affect the change of resistance. The simplified formula of coil resistance can be obtained:

(7) $$R=1.34\times 10^{-7}\frac{NR_{coil}}{d_{cu}^{2}}$$

- The number of turns, coil radius, and coil length will affect the coil inductance, and the diameter of the copper enameled wire will not affect the change of coil inductance. The simplified formula of coil inductance can be obtained:

(8) $$L=5.92\times 10^{-7}\frac{N^{2}R_{coil}}{l_{coil}}$$

The size of the coil capacitance is directly related to the size of the resonant frequency *f*. After the coil inductance value is determined, in order to expand the frequency bandwidth of the sensor, it is necessary to increase the resonant frequency and reduce the coil capacitance value as much as possible. The capacitance between layers in the coil, the capacitance between segments, the capacitance between turns, the capacitance between the coil and the magnetic core, the capacitance between the coil and the shielding layer, etc. all determine the capacitance value of the induction coil. size. It can be seen that the calculation of the coil capacitance value is a very complicated process. The arrangement of the coil, the diameter of the copper enameled wire, the winding method of the coil, etc. will all affect the coil capacitance, so it is difficult to calculate the coil capacitance value in actual operation. 

However, one thing is clear. If the coil is divided into segments, the total capacitance value can be seen as the capacitance of each segment being connected in series. This can greatly reduce the coil’s capacitance value. Therefore, the coil designed in this paper is formed by segmented winding. 

### 4.2. Noise Analysis of Magnetic Field Sensor

**Figure 11:**
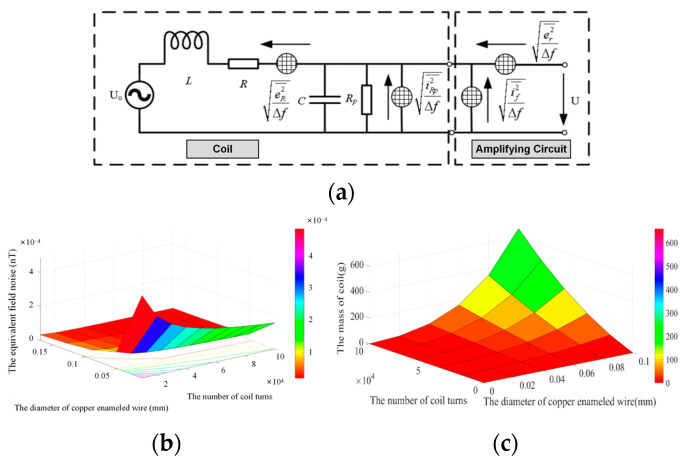
Diagram related to parameter optimization of induction coil: (**a**) Sensor noise equivalent circuit diagram; (**b**) The equivalent magnetic field noise from the magnetic field sensor; (**c**) The relationship between the coil mass, the coil parameters, and the diameter of copper enameled wire.

Sensor noise is an important part of sensor design. Noise is a useless and harmful signal, but it cannot be completely avoided. Figure 11a shows the sensor noise equivalent circuit diagram of the relevant noise source. For magnetic field measurements, the input impedance of the preamplifier should be large enough. In the circuit diagram of Figure 11a, the input impedance is negligible compared to the damping resistance *R_p_*.

Reducing noise can improve the detection accuracy and sensitivity of the sensor. The average spectral density of equivalent magnetic field noise can be written as [26]:

(9) $$\frac{\bar{H_{M}^{2}}}{\Delta f}=\frac{1}{K^{2}\cdot f^{2}}\cdot \left[\frac{1}{{\left|F\left(f\right)\right|}^{2}}\cdot \frac{e_{r}^{2}}{\Delta f}+\frac{e_{R}^{2}}{\Delta f}+\left(\frac{\bar{i_{Rp}^{2}}}{\Delta f}+\frac{\bar{i_{r}^{2}}}{\Delta f}\right)\cdot {\left|\bar{Z_{L}}\right|}^{2}\right]$$

The minimum magnetic field that can be detected by the magnetic field sensor can be determined by Formula (10). The noise expressed in Formula (10) can be regarded as white noise [27]. According to this assumption, it is reasonable to ignore the noise of 1/*f*. Therefore, it can be simplified to:

(10) $$\bar{H_{M}^{2}}=\frac{1}{K^{2}\cdot f^{2}}\left(\bar{e_{r}^{2}}+\bar{e_{R}^{2}}\right)=\bar{H_{R}^{2}}+\bar{H_{r}^{2}}$$

Typically, the choice of preamplifier determines the magnitude of $\bar{H_{r}^{2}}$. Therefore, the sensor design should reduce the noise caused by the preamplifier as much as possible. After the preamplifier is selected, if you want to further reduce the noise of the magnetic field sensor, you need to consider the thermal noise caused by the coil resistance. The resistance thermal noise produced can be expressed as:

(11) $$B_{s}=\frac{\sqrt{4kTR}}{\omega \mu_{a}NS_{h}}$$

In the formula, *k* is Boltzmann’s constant (*k* = 1.37 × 10^−23^ J/k). *T* is the thermodynamic temperature (K) (*T* = 294). *R* is the DC resistance of the induction coil, *μ_a_* is the magnetic permeability of the magnetic core, *N* is the number of turns of the coil, and *S_h_* is the cross-sectional area of the magnetic core.

The equivalent magnetic field noise of the magnetic field sensor at 100 Hz is shown in Figure 11b. When the number of turns of the coil remains constant, the diameter of the enameled copper wire increases, resulting in the decrease of the equivalent magnetic field noise of the sensor. When the diameter of the enameled copper wire remains constant, the number of turns of the coil increases, resulting in the decrease of the equivalent magnetic field noise of the sensor.

### 4.3. Coil Quality Analysis of Magnetic Field Sensors

The mass of the induction coil can be calculated as follows:

(12) $$m=\rho \times \frac{\pi^{2}d_{cu}^{2}N}{4}\left(d_{0}+\frac{Nd_{w}^{2}}{l_{coil}}\right)$$

where *ρ* is the bulk density of copper.

Figure 11c gives the relationship between the coil mass, the coil parameters, and the diameter of the copper enameled wire. When the diameter of copper enameled wire is less than 0.06 mm, the quality of the induction coil remains unchanged with the increase in the coil turns. When the diameter of copper enameled wire is greater than 0.06 mm, the mass of the induction coil increases with the increase in coil turns. 

### 4.4. Determination of Coil Parameters

The above analysis analyzes the relationship between the noise of the magnetic field sensor, the number of coil turns, the diameter of the copper enameled wire, the relationship between the coil quality and the number of coil turns, and the diameter of the copper enameled wire. Thus, the mathematical model of magnetic field sensor noise and coil quality was obtained. 

In order to obtain optimal coil parameters, you can choose to control the noise of the magnetic field sensor to a desired value, and then analyze the mathematical model of the coil quality. Therefore, this paper used the Lagrange multiplier method to calculate the optimal values of the number of coil turns and the diameter of the copper enameled wire. Through numerical calculation, it was determined that the optimal value of the coil turns is 11,000 and the optimal value of the copper enameled wire diameter is 0.08 mm, which also provided a basis for the optimal design of the magnetic field sensor. When the frequency is 100 Hz, the diameter of the enameled copper wire is *d_cu_* = 0.08 mm, and the number of turns of the coil is 11,000 turns. The equivalent magnetic field noise of the magnetic field sensor *B_s_* is 0.06 pT/Hz^1/2^, and the mass of the coil is 30 g. 

## 5. Design of Amplifier Circuit

### 5.1. Structure Design of Amplifier Circuit

**Figure 12:**
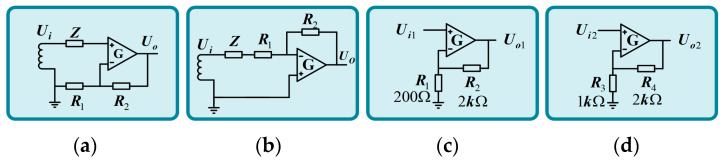
The basic circuit of the amplifier and the two-stage amplifier principle. (**a**) Non-inverting proportional amplifier. (**b**) Inverting proportional amplifier. (**c**) First-level amplification principle. (**d**) Principle of two-stage amplification.

Schematic diagrams of inverted and in-phase proportional amplifiers are shown in Figure 12a,b, where *Z* is the total impedance of the induction coil.

The output of the inverse amplifier is:

(13) $$U_{o}=-\frac{R_{2}}{R_{1}+Z}U_{i}$$

The output of the in-phase proportional amplifier is:

(14) $$U_{o}=\frac{R_{1}+R_{2}}{R_{1}}U_{i}$$

In this paper, the amplifying link of the amplifying circuit is realized by two-stage amplification, the in-phase proportional amplifier is used in each stage amplifying circuit, and the magnifying multiple is 33 times. According to this value, the parameters of the amplifying circuit are selected. In the preamplifier, *R*_1_ chooses 200 Ω resistance, and *R*_2_ uses 2 kΩ resistance. The *R*_3_ of the secondary amplifier selects a resistance of 1 kΩ, and the *R*_4_ selects a resistance of 2 kΩ, as shown in Figure 12c,d.

The overall magnification of the preamplifier circuit is:

(15) $$A_{u}=A_{u1}\cdot A_{u2}=11\times 3=33$$

**Figure 13:**
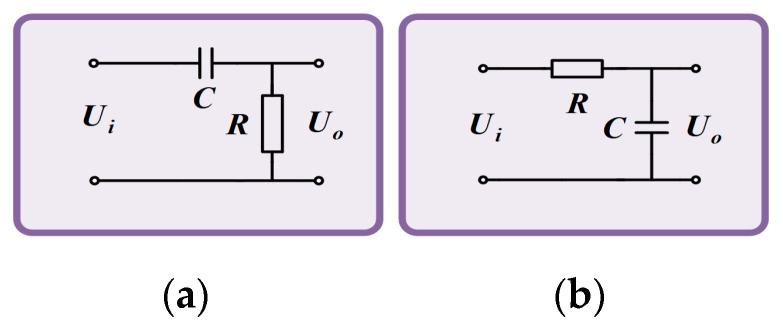
Filter circuit. (**a**) High pass filter. (**b**) Low pass filter.

In this paper, both the high-pass filter circuit and the low-pass filter circuit use the *RC* filter circuit. The *RC* filter circuit has the advantages of a simple structure and small size. The schematic diagram is shown in Figure 13.

The relationship between the output voltage *U_o_* and the input voltage *U_i_* of the high-pass filter circuit is as follows:

(16) $$U_{o}=\frac{R}{\sqrt{R^{2}+\frac{1}{4\pi^{2}f^{2}C^{2}}}}U_{i}$$

The relationship between the output voltage *U_o_* and the input voltage *U_i_* of the low-pass filter circuit is as follows:

(17) $$U_{o}=\frac{1}{2\pi fC\sqrt{R^{2}+\frac{1}{4\pi^{2}f^{2}C^{2}}}}U_{i}$$

The cutoff frequency of the *RC* filter circuit is:

(18) $$f=\frac{1}{2\pi RC}$$

To make the amplitude-frequency characteristic curve in the set frequency range more straight, the *R* of the high-pass filter circuit is 30 kΩ, the *C* is 10 μF, and the lower cut-off frequency *f_L_* of the filter link is:

(19) $$f_{L}=\frac{1}{2\pi RC}=\frac{1}{2\pi \times 30\times 10^{3}\times 10\times 10^{-6}}\approx 0.53\mathrm{Hz}$$

The *R* of the low-pass filter circuit is 3 kΩ, the *C* is 0.01 μF, and the upper cut-off frequency *f_H_* of the filter is:

(20) $$f_{H}=\frac{1}{2\pi RC}=\frac{1}{2\pi \times 3\times 10^{3}\times 0.01\times 10^{-6}}\approx 5305.16\mathrm{Hz}$$

The induction coil can be equivalent to an *RLC* series circuit. At the resonant frequency, the output voltage of the induction coil reaches the maximum value. But near this frequency, the voltage amplitude output by the induction coil is very high and the variation range is too large. If the voltage signal output by the induction coil is directly amplified near the resonant frequency, the entire amplifier circuit will produce a huge error due to the saturation effect of the integrated operational amplifier. Near the resonant frequency, the phase of the induction coil will suddenly jump from 90° to −90°. The sudden change in phase will greatly limit the frequency range of the sensor to the resonant frequency of the induction coil.

In order to improve the above situation, a magnetic flux negative feedback loop is introduced in the amplifier circuit. The induction coil generates an induced voltage in an alternating magnetic field and is first amplified by an amplifier. The amplified induced voltage is converted into a current signal through the feedback resistor. The current signal generates a magnetic field in the feedback coil in the opposite direction to the measured magnetic field, thus forming negative magnetic flux feedback. When the magnetic field is near the resonant frequency and a large voltage amplitude is output, the magnetic flux negative feedback weakens the magnetic field passing through the induction coil and reduces the induced voltage, thereby achieving the purpose of flattening the amplitude-frequency characteristic curve. After adding the feedback coil, the magnetic induction intensity through the induction coil should be *B = B_j_* − *B_f_*, and the voltage to the input of the amplifier should be:

(21) $$U_{i}=\frac{j\omega NS\mu_{a}\left(B_{j}-B_{f}\right)}{1-\omega^{2}CL_{p}+j\omega R_{L}C}$$

After the *U_i_* is magnified *G* times by the amplifier, the resulting output voltage *U_o_* is:

(22) $$U_{o}=-GU_{i}$$

The formula of feedback magnetic induction intensity is:

(23) $$B_{f}=\mu_{0}\frac{N_{f}}{d}I_{f}$$

The formula of output voltage *U_o_* is:

(24) $$U_{o}=\frac{j\omega NS\mu_{a}B_{j}G}{1-\omega^{2}CL_{p}+j\omega \left(R_{L}C-\frac{NN_{f}\mu_{a}\mu_{0}SG}{dR_{f}}\right)}$$

The transfer function between the output voltage *U_o_* and the magnetic induction intensity *B_j_* of the measured magnetic field is:

(25) $$\frac{U_{o}}{B_{j}}=\frac{j\omega NS\mu_{a}G}{1-\omega^{2}CL_{p}+j\omega \left(R_{L}C-\frac{NN_{f}\mu_{a}\mu_{0}SG}{dR_{f}}\right)}$$

The amplitude-frequency characteristics are:

(26) $$H\left(\omega \right)=\frac{\omega NS\mu_{a}G}{\sqrt{{\left(1-\omega^{2}CL_{p}\right)}^{2}+\omega^{2}{\left(R_{L}C-\frac{NN_{f}\mu_{a}\mu_{0}SG}{dR_{f}}\right)}^{2}}}$$

The phase-frequency characteristics are:

(27) $$\varphi \left(\omega \right)=90^{{}^{\circ}}-arctan\frac{\omega \left(R_{L}C-NN_{f}\mu_{a}\mu_{0}SG/dR_{f}\right)}{1-\omega^{2}CL_{p}}$$

### 5.2. Simulation of Amplifier Circuit

**Figure 14:**
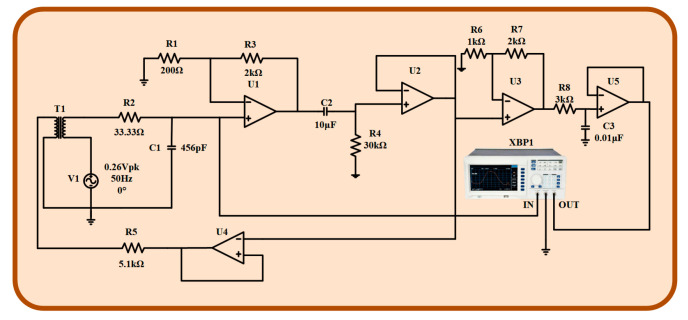
The circuit diagram of the amplifier circuit.

**Figure 15:**
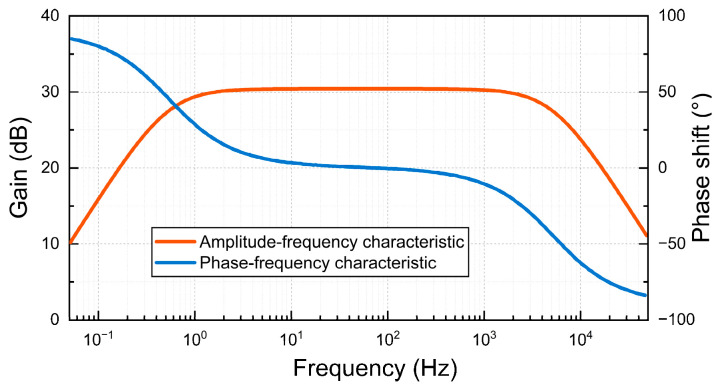
Amplitude-frequency characteristic and phase-frequency characteristic.

The simulation circuit diagram of the amplification circuit of the inductive magnetic sensor is built by using Multisim14. As shown in Figure 14, the AC voltage source V_1_ is used to simulate the induced electromotive force produced by the induction coil, and the coupled induction coil T_1_ is used to simulate the induction coil and feedback coil. The amplitude-frequency and phase-frequency characteristics of the amplifying circuit are detected by Multisim, and the amplitude-frequency characteristic curve and phase-frequency characteristic curve are obtained, as shown in Figure 15. The amplitude-frequency characteristic curve and the phase-frequency characteristic curve in the range of 10–1000 Hz are relatively flat, and the magnification meets the design requirements, which meets the design requirements of the inductive magnetic field sensor in this paper.

### 5.3. Physical Design of Amplifier Circuit

**Figure 16:**
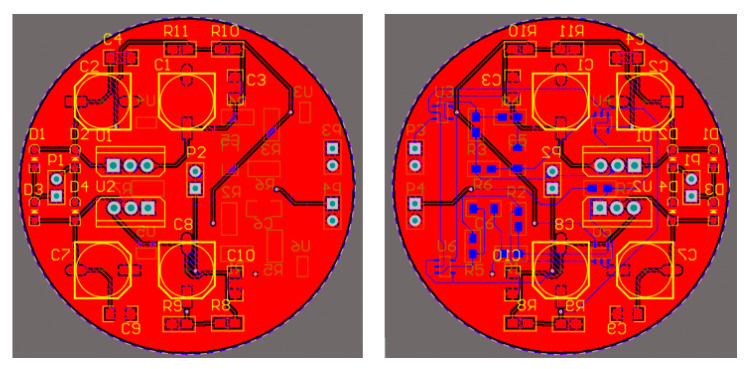
Magnifying circuit-wiring 2D-effect drawing.

**Figure 17:**
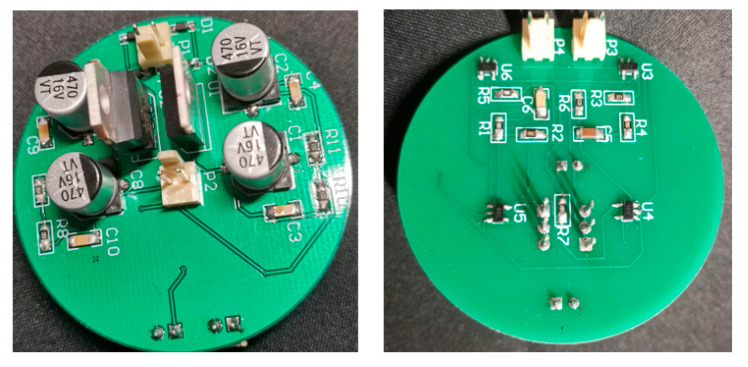
Physical diagram of the amplifier circuit.

The corresponding PCB diagram is generated according to the schematic diagram of the amplifying circuit. To reduce the volume of the magnetic field sensor, the PCB circuit board adopts a double-layer design. The top layer is the power supply circuit, and the bottom layer is the amplification circuit. The 2D effect and physical drawings of the circuit wiring are shown in Figure 16 and Figure 17.

## 6. Experimental Results

### 6.1. Test of Amplifier Circuit

The test of the circuit shows that the input of the ±2.5 V dual power-supply circuit needs to be supplied by a sinusoidal AC voltage with an amplitude of 12 V, which is provided by a transformer with a ratio of 220 to 12. The output waveforms of primary amplification and secondary amplification of the amplifying circuit are tested, respectively. As shown in Table 3, this paper shows output waveforms of several typical frequencies. When the frequency changes, whether primary amplification or secondary amplification, the output waveform keeps a complete sine wave without distortion, and no external noise is introduced. This shows that the designed amplifying circuit has high reliability.

### 6.2. Fabrication of Sensor and Calibration of Sensitivity

**Figure 18:**
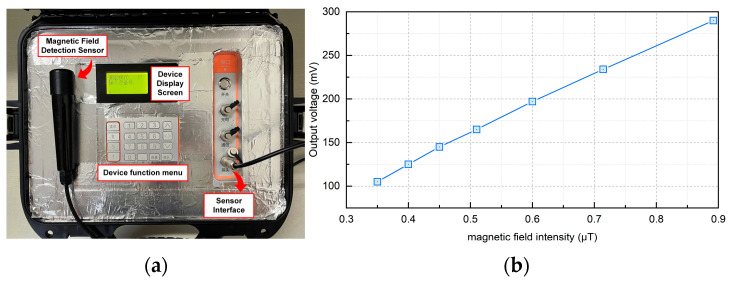
Magnetic field sensor physical object and sensitivity. (**a**) Physical diagram of magnetic field sensor. (**b**) Sensitivity of magnetic field sensor.

Figure 18a is the physical diagram of the sensor designed in this paper. The calibration of the sensor is a very important step after the manufacture of the instrument, and it plays an important role in the performance evaluation of the sensor. In particular, it is necessary to test the sensitivity of the instrument to ensure the reliability of the instrument and the accuracy and reliability of the measurement.

Considering that the interior of the electrified solenoid can provide a constant magnetic field, the electrified solenoid is selected as the device for sensitivity calibration. In this paper, the AC power supply, sliding rheostat, and electrified solenoid are connected in series to provide a variable magnetic field for the magnetic field sensor. The magnetic field sensor is placed in the electrified solenoid for calibration. Change the resistance value of the sliding rheostat to change the current through the electrified solenoid, and then, change the magnetic field inside the energized solenoid. Since the inductive magnetic field sensor is very sensitive to changing weak magnetic fields, the energized solenoid, magnetic field sensor, and calibration circuit are all placed in a magnetic field shielding tube, and the outside of the magnetic field shielding tube is grounded to ensure that during the calibration of the magnetic field sensor, it will not be disturbed by the external environment and noise. 

The sensitivity calibration curve obtained at 50 Hz is shown in Figure 18b. It can be seen that the output voltage of the magnetic field sensor and the magnetic field intensity show a good linear relationship. It is calculated that the sensitivity of the magnetic field sensor is about 327.6 mV/μT, which also means that the sensor has good performance in sensitivity and meets the current demand for cable detection in the field of power engineering. The device can achieve the reduction of volume and the improvement of portability while maintaining high precision. This feature enables the staff to use the equipment in any environment in the actual power operation and maintenance process and not have to adhere to distance, power, and other limiting factors.

### 6.3. Experimental Detection

**Figure 19:**
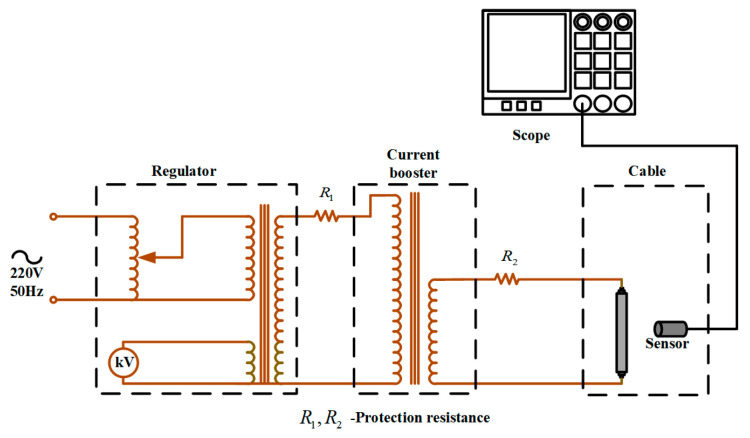
Experimental test circuit.

Figure 19 shows the experimental test platform, which consists of a power-supply system, a cable, a magnetic field sensor, and an oscilloscope. The power-supply system consists of a 220 V AC power supply, a voltage regulator, and a boost. The rated output power of the voltage regulator is 7.5 kV·A and the rated output voltage is 450 V. The voltage regulator can provide different voltages for the load and linearly adjust the voltage when it is powered on. The rated output power of the converter is 5 kV·A, the rated primary current is 20 A, and the range of output secondary current is 0–2000 A. The booster can adjust the output current steplessly to make the output current rise more balanced.

**Figure 20:**
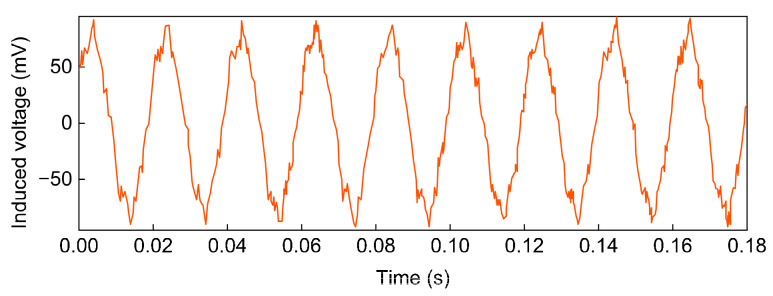
Induced voltage waveform.

The magnetic field sensor is close to the single-phase cable and displays the induced voltage waveform measured by the magnetic field sensor through the oscilloscope. By adjusting the voltage regulator, the current through the cable is set to 60 A. When the single-core cable passes through the power-frequency alternating current, the power-frequency magnetic field is bound to be generated around the cable. The voltage changes when the sensor is placed in an alternating magnetic field. The induced voltage waveform measured by the magnetic field sensor is shown in Figure 20.

The measured waveform is approximately a sine wave, which is consistent with the waveform that should appear theoretically. The waveform distortion caused by some harmonics contained in the waveform may be due to the interference of external noise and the internal problems of the cable, which is a normal phenomenon.

### 6.4. Cable Status Detection Test

The purpose of the magnetic field sensor designed in this article is to detect the operating status of the cable. Therefore, this article prepares samples of cables with different damage states. The magnetic field sensor designed in this article is then used to detect it to verify the feasibility and superiority of the design. 

**Figure 21:**
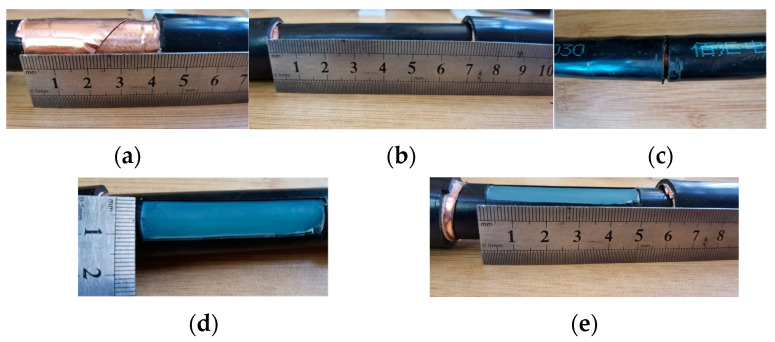
Experimental sample diagram. (**a**) Outer sheath damage. (**b**) Metal layer damage. (**c**) Wire core broken strand. (**d**) The insulation scratch is about 1 cm wide. (**e**) The insulation scratch is about 5 cm long.

First of all, the sample was prepared. Divide the cable with a total length of 15 m evenly into seven sections. Each section is about 2 m long, and these were used as experimental samples #1, #2, #3, #4, #5, and #6. The processing method for each sample is shown in Table 4, and the corresponding processing process and results are shown in Figure 21.

**Figure 22:**
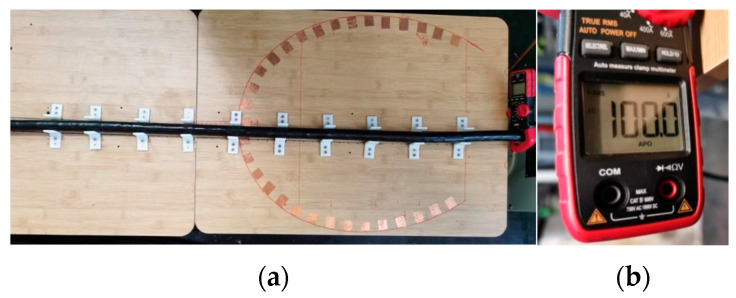
Cable magnetic field detection experiment. (**a**) Cable magnetic field detection experimental bench. (**b**) Clamp ammeter.

The detection platform is shown in Figure 22. The total length of the cable was 2 m, which was the distance connected to the two output ends of the current riser. Leave a margin of 0.5 m at each end to eliminate the influence of the connection end of the cable and the current riser on the experimental detection, and eliminate the edge effect. Therefore, the distance from the starting magnetic field detection position to the end magnetic field detection position was 1 m. The corner code was used to fix the cable to calibrate the magnetic field measurement points. The distance between the measurement points was 2 cm, and a total of 51 measurement points were calibrated. The specific experimental steps are as follows:

- Before starting the experiment, prepare different cable samples for connecting to the experimental circuit;
- Turn on the experimental power supply and adjust the voltage regulator to increase the core current to 100 A, as shown in Figure 22b;
- For samples in different states, a magnetic field sensor is used to detect the magnetic field signals at the measurement points. During detection, the distance between the sensor probe and the cable is 2 mm; that is, the vertical distance between the probe and the cable surface is maintained at 2 mm. Store the waveform of the acquisition point into the status information database of the corresponding state.

**Figure 23:**
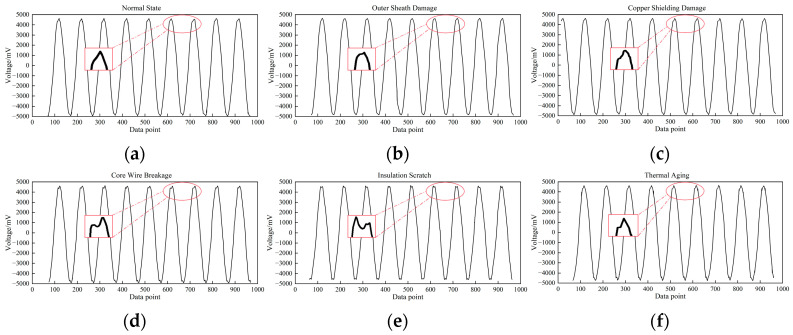
Experimental test results. Magnetic field waveforms of different cable conditions. (**a**) Normal state. (**b**) Outer sheath damage. (**c**) Copper-shielding damage. (**d**) Core wire breakage. (**e**) Insulation scratch. (**f**) Thermal aging.

The detection results are shown in Figure 23. Figure 23 includes the result of the cable in the defect-free state and the cable inspection results in five different defect states. The magnetic field signal usually presents a good sinusoidal waveform. The signal difference between the six cable conditions is mainly concentrated in the distortion at the peak of the waveform. From the test results, it is obvious that the magnetic field sensor designed in this paper has higher detection accuracy.

## 7. Conclusions

Based on the electromagnetic characteristics of an AC power cable, this paper optimizes the design and development of an AC power-cable magnetic field detection sensor. The main conclusions are as follows:

1. Permalloy has enough initial permeability and good ductility. The price is relatively cheaper, but it also has good thermal stability. So, permalloy is selected as the material of the magnetic core in this paper;
2. The geometric parameters of the magnetic core are optimized by the simulation model, and the optimal aspect ratio of the magnetic core is determined to be 20;
3. The smaller the ratio of coil length to core length, the higher the sensitivity of the sensor. After analysis, the ratio of coil length to core length is set to 0.3;
4. When the diameter of the copper enameled wire is 0.08 mm and the number of turns of the coil is 11,000, the equivalent magnetic field noise of the sensor is 0.06 pT, and the mass of the coil is only 30 g;
5. In this paper, the magnetic flux negative feedback link is introduced into the amplifying circuit, which greatly improves the transmission characteristics of the sensor, makes the amplitude-frequency characteristic and phase-frequency characteristic curve smoother, and expands the frequency bandwidth of the magnetic field sensor. Verified by simulation and experimental tests, the amplifying circuit has high reliability.

In this paper, a new type of non-contact magnetic field sensor is designed, which is small and easy for measuring personnel to use. It can be used for the rapid detection of power cables and the timely detection and repair of power cable faults. It is of great significance to maintain the safe and stable operation of the power grid.

## Figures and Tables

**Table 1:** Introduction of magnetic field measurement methods.

Principle of Measurement	Example	Range of Measurement (T)	Application
Magnetic torque	Geomagnetic variometer	10^−11^~10^−7^	Earth’s magnetic field
Fluxgate	Peak detector	10^−9^~10^−1^	Weak magnetic field
Electromagnetic induction	Fixed coil	10^−10^~10^−1^	Alternating magnetic field
Electromagnetic effect	Hall transducer	10^−7^~10	Pulsed magnetic field
Magneto-optical effect	Faraday magneto-optical effect	10^−2^~10	The laser device

**Table 2:** Representative parameters of magnetic core materials.

Materials	B_s_/T	μ_i_	μ_m_	ρ/μΩ × cm	Applicable Frequency Range
Mn-Zn ferrite	0.38	3000	12,000	1–10	Tens of MHz
Permalloy	0.74	50,000	150,000	55	<20 kHz
Iron-based amorphous alloy	1.56	50,000	200,000	130	50 Hz–10 kHz
Nanocrystalline iron-based alloy	1.45	100,000	800,000	115	<500 kHz

**Table 3:** The output waveform of the amplifier circuit when the frequency changes.

Typical Frequency	Primary Amplification	Secondary Amplification
10 Hz	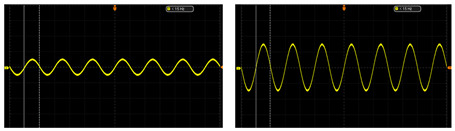	
50 Hz	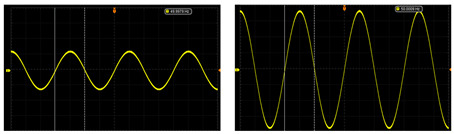	
500 Hz	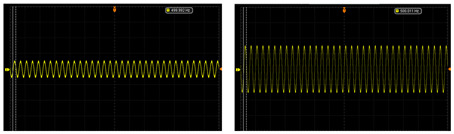	
1000 Hz	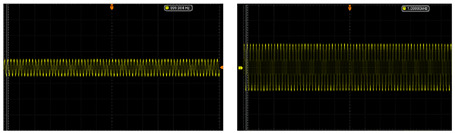	

**Table 4:** Experimental sample processing methods.

Sample	Treatment Method
#1	Without any treatment, as a control sample.
#2	Make a damaged outer sheath in the middle of the cable and peel off the outer sheath 4 cm.
#3	Make a damage defect in the metal sheath in the middle of the cable, and peel off the metal sheath 7 cm.
#4	Make a broken core strand in the middle of the cable and use an electric saw to cut the sample until the core is partially broken.
#5	Create scratch defects in the insulation layer in the middle of the cable.
#6	Thermal aging treatment is to place the sample in a constant temperature aging box to thermally age the sample. The aging temperature is 90 °C and the aging time is 70 days.

## Data Availability

Data are contained within the article.
